# Objective Measurement of Physical Activity and Sedentary Behavior Among US Adults Aged 60 Years or Older

**Published:** 2011-12-15

**Authors:** Kelly R. Evenson, David M. Buchner, Kimberly B. Morland

**Affiliations:** University of North Carolina, Gillings School of Global Public Health, Department of Epidemiology; University of Illinois at Urbana-Champaign, Champaign, Illionois; Mount Sinai School of Medicine, New York, New York

## Abstract

**Introduction:**

Estimates of objectively measured physical activity among older adults differ depending on the cut points used to define intensity. Our objective was to assess 1) moderate to vigorous physical activity (MVPA), exploring differences in prevalence depending on the cut point used, and 2) sedentary behavior, among US adults aged 60 years or older.

**Methods:**

We used the most recently available accelerometer data (2003-2004 and 2005-2006) from the National Health and Nutrition Examination Survey. The sample included 2,630 adults aged 60 or older who wore an ActiGraph accelerometer for 1 week. We defined MVPA using 4 cut points (≥500, ≥1,000, ≥1,500, and ≥2,000 counts/min) and sedentary behavior as fewer than 100 counts per minute. All estimates were weighted to reflect the US population.

**Results:**

The overall average counts per minute was 216. Mean MVPA ranged from 10.8 minutes per day (based on 2,000 counts/min) to 106.8 minutes per day (based on 500 counts/min). Regardless of the cut point used, MVPA was lower with each successive age group (60-69, 70-79, and ≥80) and higher among men than women. At any cut point, MVPA was highest for Hispanics and "other" racial/ethnic groups, followed by non-Hispanic whites and non-Hispanic blacks. The average time spent in sedentary behavior was 8.5 hours per day. The amount of sedentary time was greater among participants who were aged 80 or older compared to younger groups and among men compared to women.

**Conclusion:**

MVPA estimates vary among adults aged 60 or older, depending on the cut point chosen, and most of their time is spent in sedentary behaviors. These considerations and the data presented suggest more research is necessary to identify the appropriate method of setting accelerometer cut points for MVPA in older adults.

## Introduction

Self-reported data from the 2005-2006 National Health and Nutrition Examination Survey (NHANES) indicate that 60% to 63% of US adults aged 60 to 69 years and 47% to 51% of adults aged 70 or older met current US recommendations for physical activity (1,2). Prevalence estimates for the same population are much lower, however, if based on objectively measured physical activity using accelerometry; only 9% to 26% of adults aged 60 to 69 and 6% to 10% of adults aged 70 or older met current US recommendations for physical activity (2). This discrepancy may be due to overreporting of physical activity because of difficulty recalling activities, cultural differences in interpretation, social desirability bias, or the way the questions are worded (3).

Accelerometry is a way to objectively assess physical activity that overcomes many of the limitations of self-report. An accelerometer is an instrument worn by study participants; it detects acceleration in selected planes and converts the data into "counts," which are then measured in specific time intervals or epochs. Higher counts result from greater acceleration. Researchers often rely on calibration studies to determine the appropriate count cut point or cut points to identify intensity levels (4) of sedentary, light, moderate, or vigorous physical activity. However, calibration studies report a wide range of cut points to define MVPA for older adults. For example, using the ActiGraph accelerometer, studies that included adults aged 60 or older recommended MVPA cut points ranging from 574 to 2,020 counts per minute (3,5-7). One reason for the wide ranges is that some calibration studies focus mainly on ambulation to determine cut points (3,6,7), but others include lifestyle activities (5). It is unclear to what degree the difference in cut points may alter the reported prevalence of physical activity.

Another intriguing finding of the 2005-2006 NHANES was that similar proportions of Hispanic adults (9%-10%) and non-Hispanic white adults (8%-10%) met physical activity guidelines when measurements were based on accelerometry (2); however, self-reported findings from the same populations indicated lower levels of physical activity among Hispanics (41%-44%) compared to non-Hispanic whites (63%-65%). It is unclear why the differences between self-report and objectively measured physical activity by race/ethnicity persist with older age groups. Sedentary behavior among older adults has also not been well described.

The objective of this study was to use nationally representative data from NHANES for adults aged 60 or older to 1) describe MVPA and explore differences in prevalence depending on the cut point used to define MVPA and 2) describe sedentary behavior. For both aims, we report prevalence overall and by age group, sex, and race/ethnicity. To increase the stability of the estimates, we combined data from 2003 to 2006, the most recent accelerometer data available.

## Methods

NHANES uses a complex, multistage probability sample of the civilian US noninstitutionalized population of all ages. To produce reliable estimates, the survey oversamples several population groups, including adults aged 60 or older. NHANES conducts in-home interviews, in English or Spanish, using a computer-assisted personal interview system, and physical examinations at a mobile examination center. The unweighted response rates for the interviews were 79% (2003-2004) and 81% (2005-2006) and for the examinations were 76% (2003-2004) and 78% (2005-2006) (8-11).

### Objectively measured physical activity

NHANES used the ActiGraph model AM7164 (ActiGraph, LLC, Fort Walton Beach, Florida) to measure acceleration. The instrument converted acceleration sampled multiple times to a digital signal, which was stored as a count. The ActiGraph is a technically reliable instrument, able to detect differing levels of intensity (12,13). From 2003 to 2006, ambulatory participants aged 6 years or older received an accelerometer at their examination to wear for 7 consecutive days (14). The instrument recorded 1-minute intervals starting at midnight after the examination. It was strapped to an elastic belt and worn over the right hip. It was not waterproof, so participants were asked to remove the monitor when showering or engaging in water activities such as swimming. They were also told to remove it at bedtime. Participants received $40 upon return of the accelerometer by mail.

Participants did not keep a diary of when the monitor was put on and taken off. Nonwear time was defined as a period of 60 minutes or more when the accelerometer recorded zeros, with allowance for 1 or 2 minutes of counts between 0 and 100, as recommended elsewhere (7,15). Counts for nonwear minutes were set to missing, as were those for spurious data, defined either as counts per minute of at least 30,000 or at least 10 minutes of the same repeated nonzero counts.

We examined the accelerometer data in 3 ways. First, using mean counts per minute, we evaluated the raw data, which represented average intensity of physical activity without imposing cut point decisions. Second, we determined levels of MVPA by applying a range of cut points inclusive of recommendations in the literature (3,5-7): a minimum of 500, 1,000, 1,500, or 2,000 counts per minute. We classified sedentary behavior as fewer than 100 counts per minute (16). Third, bouts of MVPA were defined as periods of at least 10 consecutive minutes above the cut point, with allowance for interruptions of 1 to 2 minutes below the cut point, similar to other reports (2,7).

### Self-reported measures

NHANES questionnaires, protocols, and coding for the self-reported data are described elsewhere (www.cdc.gov/nchs/nhanes.htm). All participants who were aged 85 or older were assigned an age of 85 before the data were released to protect their confidentiality. Other self-reported measures used to describe the participants included race/ethnicity, education, household income, marital status, current smoking (defined as use of cigarettes in the past 5 days), and limitations to activity assessed by 3 questions. Participants were asked if a physical, mental, or emotional problem kept them from working, if these problems limited the kind of work they did, or if they had difficulty walking without using any special equipment. Responses to each of the 3 questions were yes or no.

The questionnaire also assessed physical activity; it defined moderate-intensity physical activity as tasks that caused light sweating or a slight to moderate increase in breathing or heart rate and vigorous-intensity physical activity as tasks that caused heavy sweating or large increases in breathing or heart rate. Participants reported separately about past-month moderate and vigorous leisure activities, describing each by frequency, duration, and type of activity.

### Statistical analyses

NHANES included 3,471 participants who were 60 or older for 2 study periods, 2003-2004 and 2005-2006. Of these, 561 did not wear the accelerometer, 140 returned an accelerometer that was out of calibration, and 140 did not provide at least 3 compliant days (Appendix A), leaving 2,630 adults for the analysis (1,418 in 2003-2004 and 1,212 in 2005-2006).

For all analyses, we weighted all percentages and means using sample weights to account for the differential probability of selection. We calculated 4-year sample weights from the base probabilities of selection, adjusted for nonresponse to participation in the NHANES physical activity monitor component (by modifying this SAS program: http://riskfactor.cancer.gov/tools/nhanes_pam/reweight.html) and poststratified the data to match population total controls (9,10). The data were nested (ie, screener, household interview, examination), so we adjusted for nonresponse and poststratification. To compare the effect of differing cut points, we calculated results overall and stratified by age group (60-69, 70-79, ≥80), sex, and race/ethnicity (non-Hispanic white, non-Hispanic black, Hispanic, and "other"). We assessed selection bias by comparing 2 independent samples with the χ^2^ tests for categorical variables on unweighted data; significance was set at *P* < .10. We conducted analyses using SAS version 9.2 (SAS Institute, Inc, Cary, North Carolina).

## Results

### Sample

Participants who met inclusion criteria (n = 2,630) were more likely than participants with incomplete accelerometer data (n = 841) to be younger, be Hispanic, have higher education, be married, not report limitations that kept them from working, not use special equipment for walking, and self-report meeting physical activity recommendations. Among the participants, the mean age was 71.6 (median, 71.0), and 56.1% were women (Table 1). Most (82.5%) were non-Hispanic white. Nearly half (44.6%) had education beyond high school, and 26.0% had less than a high school education. Overall, 15.8% reported needing special equipment to walk because of a health problem.

### Moderate to vigorous physical activity

Participants averaged 216.4 counts per minute (Table 2). The mean number of minutes per day of MVPA ranged from 10.8 (cut point ≥2,000 counts/min) to 106.8 (cut point ≥500 counts/min) (Table 2). Time spent in bouts of at least 10 minutes of MVPA (allowing for up to 2 minutes to fall below the cut point) ranged from an average of 9.2 (based on cut point ≥2,000 counts/min) to 59.0 minutes per day (cut point ≥500 counts/min) (Table 3). Among participants who engaged in at least 1 bout, the mean amount of time in bouts of at least 10 minutes was greater: 27.6 minutes per day (cut point ≥2,000 counts/min [n = 731]) to 66.1 minutes per day (cut point ≥500 counts/min [n = 2,279]) (Appendix B). MVPA and number of minutes in bouts of MVPA were lower with each successive age group, regardless of the cut point used. The 60- to 69-year-old group ranged from 14.2 minutes per day (cut point ≥2,000 counts/min) to 130.7 minutes per day (cut point ≥500 counts/min) of MVPA (Table 2). In contrast, the oldest age group ranged from 5.6 minutes per day (cut point ≥2,000 counts/min) to 64.0 minutes per day (cut point ≥500 counts/min) of MVPA. MVPA and number of minutes in bouts of MVPA were higher among men than women, using any of the 4 MVPA cut points (Tables 2 and 3). Differences in minutes per day were more pronounced at the highest cut point (≥2,000 counts/min). Among participants with at least 1 bout, the mean amount of time in bouts of at least 10 minutes was greater for men than women, using all 4 cut points (Appendix B). Using any of the 4 MVPA cut points, Hispanics or people classified as "other" race/ethnicity consistently had the highest level of MVPA or minutes spent in bouts of MVPA, followed by non-Hispanic whites and non-Hispanic blacks.

### Sedentary behavior

The average amount of time spent in sedentary behavior was 8.5 hours per day (Table 4). Sedentary time was greatest among participants who were aged 80 or older compared to younger age groups. Women spent less time in sedentary behaviors compared to men, and Hispanics were least sedentary compared to other racial/ethnic groups.

## Discussion

The cut point used to define MVPA affected the number of minutes of MVPA among US adults aged 60 or older, and thus the calculation of the prevalence of meeting physical activity recommendations. Using 2003-2004 NHANES data at a cut point of at least 2,020 counts per minute, one study found that MVPA among 60- to 69-year-olds averaged 12.4 minutes per week for women and 16.7 minutes per week for men (7). For adults aged 70 or older, the same study (7) reported 5.4 minutes per week for women and 8.7 minutes per week for men. In contrast, using 2003-2006 NHANES data with a range of cut points, we found that the number of minutes per day of MVPA for participants aged 60 to 69 ranged from 11.4 to 124.1 for women and 17.4 to 138.2 for men, and for participants aged 70 or older ranged from 5.7 to 92.2 for women and 5.5 to 103.8 for men.

The use of fixed cut points to determine MVPA may be most problematic for older adults. For some older adults, activities above the cut point of 6 metabolic equivalents (METS) that generally is used to indicate vigorous activity are not possible because of their declining maximal cardiorespiratory fitness. On average, for adults aged 65 to 79, moderate-intensity activity perceived as "somewhat hard" corresponds to 3.2 to 4.7 METS, and vigorous-intensity activity perceived as "hard" corresponds to 4.8 to 6.7 METS (17). For adults aged 80 or older, moderate-intensity activity perceived as "somewhat hard" corresponds to 2.0 to 2.9 METS, and vigorous-intensity activity perceived as "hard" corresponds to 3.0 to 4.3 METS.

The range in walking and running speeds for older adults, who have lower fitness levels, is smaller than for younger adults. This smaller range in activities can pose problems for the development of optimal cut points for different intensities of activities (3). As cardiorespiratory fitness declines, the relative intensity of effort required for a given activity changes significantly (18). This change in effort may result, for example, in performing light activities (defined on an absolute scale requiring <3 METS) using moderate effort. Older adults may also have altered gait patterns (19) and a higher metabolic "cost" of walking (20), both of which can affect the cut point used in accelerometry to determine MVPA. An accelerometer cut point that is set too high will result in an underestimation of MVPA.

The national physical activity recommendations acknowledge the importance of relative-intensity physical activity for older adults, especially those with lower functional capacities (1,21,22). Relative intensity can account for cardiorespiratory fitness to assess effort, whereas absolute intensity is based on the rate of energy expended. This approach is supported by findings of the Harvard Alumni Health Study that in older men, relative-intensity physical activity was a stronger predictor of coronary heart disease than absolute-intensity physical activity (18).

Because of the challenges in measuring MVPA, Pruitt et al (4) explored an alternative way to determine moderate-intensity cut points for physical activity. They asked 93 participants aged 70 to 89 to walk 400 meters at their usual pace while wearing an ActiGraph accelerometer. After 4 laps, participants were asked how they perceived intensity. They were told to continue walking at that pace if at a "light" or "somewhat hard" intensity but to slow down if they perceived the walking to be "hard" or "very hard." The cut points generated for MVPA varied and ranged from 149 to 3,133 counts per minute, with a weighted average of 1,456 counts per minute for the group. The authors concluded that to differentiate activity levels in their sample of older adults, individualized cut points were more effective than group-level cut points. Given the lack of consistent MVPA cut points to use for older adults, an individualized approach may be necessary to determine the correct cut point, especially among people with low cardiorespiratory fitness. This method could use relative intensity to help determine whether physical activity recommendations have been met.

Separately, we found that the patterns of MVPA were consistent among age, sex, and race/ethnicity groups, regardless of cut point. Generally, self-report measures indicate that in the United States, white adults are more active than other racial/ethnic groups (23). By contrast, we found that among adults aged 60 or older, overall activity was higher among Hispanics and other races/ethnicities. The difference between the self-report and objective measures may be due to cultural differences in interpretation or to the types of activities, something that cannot yet easily be distinguished with the accelerometer used in this study.

This study has several limitations. First, the study sample differed from NHANES participants who were excluded because of accelerometer noncompliance by several demographic and disability characteristics. Second, the accelerometer used in NHANES records ambulatory activity well, but can underestimate activities such as bicycling, climbing stairs, lifting weights, or carrying loads. The accelerometer readings may also vary among people who use assistive devices to walk. Third, participants were told not to wear the monitor during water activities, such as swimming. Thus, all of these activities are underrepresented in the accelerometer data.

This study provides national estimates of objectively measured physical activity among adults aged 60 or older. MVPA was higher with each successive age group, by at least 2-fold regardless of the MVPA cut point, between 60- to 69-year-olds and participants who were aged 80 or older. MVPA was higher among men than women, using any of the 4 MVPA cut points, with larger gaps at the higher intensity levels. Hispanics and "other" racial/ethnic groups had higher levels of MVPA using any of the 4 cut points, followed by non-Hispanic whites and non-Hispanic blacks. These considerations and the data presented suggest more research is necessary to identify the appropriate method of setting accelerometer cut points for MVPA in older adults. The NHANES estimates of MVPA in older adults vary substantially depending upon the choice of accelerometer cut point.

## Figures and Tables

**Table 1:** Characteristics of Adults Aged 60 Years or Older (n = 2,630), NHANES, 2003-2006

**Characteristic**	% (95% CI)^a^
**Age, y**	
60-69	47.3 (44.4-50.2)
70-79	33.9 (31.5-36.3)
≥80	18.8 (16.8-20.9)
**Sex**	
Male	43.9 (41.7-46.1)
Female	56.1 (53.9-58.3)
**Race/ethnicity**	
Non-Hispanic white	82.5 (78.4-86.7)
Non-Hispanic black	8.6 (6.1-11.1)
Hispanic	5.2 (3.0-7.4)
Other	3.7 (2.8-4.6)
**Education**	
Less than high school	26.0 (21.9-30.1)
High school graduate or equivalent	29.4 (27.0-31.8)
Greater than high school	44.6 (40.3-48.9)
**Annual household income, $**	
<15,000	15.2 (12.7-17.7)
15,000-34,999	36.5 (32.9-40.1)
35,000-64,999	29.0 (25.9-32.0)
≥65,000	19.3 (15.3-23.3)
**Married**	60.3 (57.2-63.5)
**Behaviors and conditions**	
Cigarette smoking past 5 days	11.9 (10.1-13.6)
Physical, mental, or emotional problem keeps them from working at a job	16.8 (14.2-19.4)
Physical-mental, or emotional problem limits the kind or amount of work they do	35.1 (32.0-38.2)
Have difficulty walking without using any special equipment because of a health problem	15.8 (13.7-17.8)
Meet recommendation for physical activity^b^	25.3 (22.6-28.1)

Abbreviations: NHANES, National Health and Nutrition Examination Survey; CI, confidence interval.

a Values are weighted.

b Adults were classified as meeting recommendations if they reported at least 150 min/wk of moderate-intensity activity, at least 75 min/wk of vigorous-intensity activity, or an equivalent combination of the 2.

**Table 2:** Counts per Minute and Minutes per Day of Physical Activity, Overall and by Age, Sex, and Race/Ethnicity, NHANES, 2003-2006

Characteristic	n	Counts/Min, Mean (95% CI)	Min/Day, Mean (95% CI)^a^			

			Cut Point of ≥500 Counts/Min	Cut Point of ≥1,000 Counts/Min	Cut Point of ≥1,500 Counts/Min	Cut Point of ≥2,000 Counts/Min
**Overall**						
**Overall**	2,630	216.4 (207.6-225.2)	106.8 (102.0-111.7)	42.3 (39.2-45.3)	20.0 (18.0-22.0)	10.8 (9.5-12.2)
**Age, y**						
60-69	1,186	256.2 (245.8-266.5)	130.7 (125.0-136.5)	54.6 (51.4-57.9)	26.2 (24.4-28.1)	14.2 (13.0-15.5)
70-79	861	198.1 (185.7-210.4)	97.2 (90.5-103.9)	36.7 (32.2-41.1)	17.0 (13.7-20.2)	9.0 (6.8-11.3)
≥80	583	149.7 (128.2-171.2)	64.0 (56.6-71.3)	21.3 (16.1-26.6)	9.9 (6.0-13.8)	5.6 (2.1-9.1)
**Sex**						
Male	1,316	231.3 (220.9-241.7)	115.6 (110.3-121.0)	50.2 (46.3-54.0)	24.8 (22.0-27.7)	13.6 (11.6-15.5)
Female	1,314	204.7 (195.7-213.7)	99.9 (94.9-105.0)	36.1 (33.4-38.8)	16.2 (14.6-17.9)	8.7 (7.4-10.0)
**Race/ethnicity**						
Non-Hispanic white	1,578	216.6 (206.9-226.2)	106.6 (101.3-111.9)	42.2 (38.8-45.6)	20.1 (17.8-22.4)	11.0 (9.4-12.5)
Non-Hispanic black	445	193.0 (180.0-206.0)	95.5 (85.9-105.2)	35.1 (30.9-39.2)	15.0 (13.1-17.0)	7.2 (6.1-8.4)
Hispanic	530	246.9 (228.4-265.5)	122.9 (114.2-131.6)	50.7 (44.9-56.5)	24.1 (20.6-27.7)	12.7 (10.5-14.8)
Other	77	224.7 (189.8-259.6)	115.8 (90.3-141.3)	48.1 (34.9-61.3)	24.3 (17.8-30.8)	14.3 (10.7-17.9)
**Age 60-69 y**						
**Sex**						
Male	569	271.9 (258.8-285.0)	138.2 (131.6-144.8)	62.6 (58.8-66.3)	31.5 (29.2-33.7)	17.4 (15.8-19.0)
Female	617	242.2 (231.3-253.0)	124.1 (117.5-130.8)	47.6 (44.1-51.1)	21.6 (19.6-23.6)	11.4 (10.1-12.6)
**Race/ethnicity^b^**						
Non-Hispanic white	570	257.1 (244.8-269.3)	131.1 (124.3-137.8)	54.8 (51.0-58.6)	26.4 (24.1-28.6)	14.3 (12.8-15.8)
Non-Hispanic black	265	224.3 (212.2-236.5)	113.1 (104.5-121.8)	44.0 (39.9-48.1)	19.3 (17.3-21.3)	9.5 (8.1-10.9)
Hispanic	307	281.2 (255.2-307.3)	142.2 (132.7-151.8)	61.2 (54.6-67.8)	29.7 (25.7-33.7)	15.7 (13.0-18.5)
**Age 70-79 y**						
**Sex**						
Male	469	209.2 (191.5-226.9)	103.8 (95.6-111.9)	43.8 (36.5-51.2)	21.5 (15.4-27.6)	11.6 (7.5-15.8)
Female	392	189.5 (177.0-202.0)	92.2 (84.7-99.8)	31.2 (27.3-35.0)	13.4 (11.5-15.4)	7.1 (5.8-8.3)
**Race/ethnicity^b^**						
Non-Hispanic white	538	200.8 (186.7-214.8)	98.4 (91.0-105.8)	37.5 (32.4-42.6)	17.6 (13.8-21.4)	9.5 (6.9-12.1)
Non-Hispanic black	136	170.6 (156.3-184.8)	84.7 (72.9-96.5)	28.1 (22.8-33.5)	11.2 (8.6-13.8)	5.1 (3.8-6.3)
Hispanic	167	221.7 (199.6-243.8)	111.9 (97.1-126.7)	43.9 (35.2-52.7)	20.2 (14.5-25.8)	10.2 (7.1-13.3)
**Age ≥80 y**						
**Sex**						
Male	278	148.7 (140.4-156.9)	68.7 (62.7-74.8)	23.9 (21.3-26.5)	10.7 (8.9-12.5)	5.5 (4.1-6.8)
Female	305	150.3 (116.4-184.1)	61.2 (50.0-72.4)	19.8 (11.6-28.1)	9.5 (3.3-15.6)	5.7 (0.2-11.2)
**Race/ethnicity^b^**						
Non-Hispanic white	470	151.1 (127.5-174.6)	64.6 (57.1-72.2)	21.6 (15.8-27.4)	10.1 (5.7-14.5)	5.8 (1.8-9.8)
Non-Hispanic black	44	136.3 (109.3-163.4)	60.0 (41.6-78.3)	20.1 (13.7-26.5)	8.8 (6.0-11.5)	4.3 (2.8-5.8)
Hispanic	56	161.7 (137.3-186.2)	67.1 (52.8-81.4)	22.2 (15.3-29.2)	10.2 (5.9-14.6)	5.7 (2.4-9.1)

Abbreviations: NHANES, National Health and Nutrition Examination Survey; CI, confidence interval.

a Values are weighted. Counts per minute were measured on accelerometers worn by participants. Cut points of counts per minute indicate intensity of physical activity.

b The "other" race/ethnicity category is not reported when stratifying by age because of the small sample size. However, it was included as part of the calculation.

**Table 3:** Minutes per Day of Physical Activity in Bouts^a^, Overall and by Age, Sex, and Race/Ethnicity, NHANES, 2003-2006

Characteristic	n	Mean (95% CI)^b^			

		Cut Point of ≥500 Counts/Min	Cut Point of ≥1,000 Counts/Min	Cut Point of ≥1,500 Counts/Min	Cut Point of ≥2,000 Counts/Min
**Overall**	2,630	59.0 (54.7-63.4)	24.2 (21.1-27.3)	13.8 (11.5-16.1)	9.2 (7.2-11.3)
**Age, y**					
60-69	1,186	73.7 (68.5-78.8)	30.1 (27.0-33.1)	17.0 (14.9-19.2)	11.1 (9.4-12.8)
70-79	861	51.5 (46.0-57.1)	21.2 (16.9-25.4)	12.1 (8.5-15.7)	7.9 (5.3-10.6)
≥80	583	35.9 (27.1-44.7)	14.9 (6.1-23.6)	8.8 (1.4-16.1)	6.8 (−1.2-14.8)
**Sex**					
Male	1,316	68.2 (62.8-73.6)	29.3 (25.1-33.6)	16.7 (13.6-19.9)	10.3 (8.2-12.4)
Female	1,314	51.9 (47.2-56.5)	20.1 (16.8-23.5)	11.5 (8.8-14.2)	8.4 (5.4-11.3)
**Race/ethnicity**					
Non-Hispanic white	1,578	57.4 (52.6-62.1)	24.2 (20.6-27.8)	14.1 (11.5-16.7)	9.5 (7.2-11.8)
Non-Hispanic black	445	55.7 (48.9-62.6)	16.5 (14.3-18.6)	8.1 (6.7-9.6)	5.3 (4.0-6.6)
Hispanic	530	75.8 (65.2-86.3)	26.6 (23.1-30.2)	14.8 (12.2-17.4)	9.4 (7.3-11.5)
Other	77	80.7 (54.0-107.3)	38.6 (26.2-50.9)	19.8 (14.4-25.3)	12.9 (8.8-17.0)

Abbreviations: NHANES, National Health and Nutrition Examination Survey; CI, confidence interval.

a Bouts were defined as ≥10 consecutive minutes above the cut point, with allowance for interruptions of 1 to 2 minutes below the cut point.

b Values are weighted. Counts per minute were measured on accelerometers worn by participants. Cut points of counts per minute indicate intensity of physical activity.

**Table 4:** Minutes per Day of Sedentary Behavior^a^, Overall and by Age, Sex, and Race/Ethnicity, NHANES, 2003-2006

**Characteristic**	n	Mean (95% CI)^b^
**Overall**		
**Overall**	2,630	507.7 (501.3-514.1)
**Age, y**		
60-69	1,186	487.8 (477.0-498.6)
70-79	861	516.2 (506.8-525.6)
≥80	583	542.4 (532.2-552.6)
**Sex**		
Male	1,316	519.2 (509.4-529.0)
Female	1,314	498.7 (491.9-505.6)
**Race/ethnicity**		
Non-Hispanic white	1,578	507.6 (500.1-515.2)
Non-Hispanic black	445	516.7 (504.7-528.6)
Hispanic	530	479.4 (461.5-497.2)
Other	77	528.7 (502.8-554.6)
**Age 60-69 y**		
**Sex**		
Male	569	500.5 (485.4-515.6)
Female	617	476.5 (464.9-488.1)
**Race/ethnicity^c^**		
Non-Hispanic white	570	487.6 (474.9-500.3)
Non-Hispanic black	265	494.1 (483.1-505.2)
Hispanic	307	462.8 (438.0-487.5)
**Age 70-79 y**		
**Sex**		
Male	469	527.1 (514.1-540.2)
Female	392	507.8 (494.8-520.8)
**Race/ethnicity^c^**		
Non-Hispanic white	538	514.1 (503.6-524.6)
Non-Hispanic black	136	530.7 (509.6-551.8)
Hispanic	167	498.4 (472.5-524.4)
**Age ≥80 y**		
**Sex**		
Male	278	561.9 (547.9-576.0)
Female	305	531.0 (517.6-544.4)
**Race/ethnicity^c^**		
Non-Hispanic white	470	542.1 (533.4-550.8)
Non-Hispanic black	44	562.1 (516.1-608.0)
Hispanic	56	503.6 (474.2-533.1)

Abbreviations: NHANES, National Health and Nutrition Examination Survey; CI, confidence interval.

a Sedentary behavior is defined as <100 counts/min, as measured on accelerometers worn by participants.

b Values are weighted.

c The "other" race/ethnicity category is not reported when stratifying by age because of the small sample size. However, it was included as part of the calculation.

## Appendices

### Appendix A. Study inclusion criteria and accelerometer analysis

http://www.unc.edu/~kevenson/_Evenson_2012_PCD_AppendixA.pdf

### Appendix B. Minutes per day of physical activity in bouts, overall and by age, sex, and race/ethnicity, among participants with at least one bout, NHANES, 2003-2006

http://www.unc.edu/~kevenson/_Evenson_2012_PCD_AppendixB.pdf
